# Context dependency, co-introductions, novel mutualisms, and host shifts shaped the ectomycorrhizal fungal communities of the alien tree *Eucalyptus globulus*

**DOI:** 10.1038/s41598-019-42550-x

**Published:** 2019-05-09

**Authors:** Serena Santolamazza-Carbone, Mónica Durán-Otero, María Calviño-Cancela

**Affiliations:** 1 University of Vigo, EUET Forestal, Department of Ecology and Animal Biology, Campus A Xunqueira, 36005 Pontevedra, Spain; 2 University of Vigo, Faculty of Sciences, Department of Ecology and Animal Biology, Campus Lagoas-Marcosende, 36310 Vigo, Spain

**Keywords:** Ecology, Forestry

## Abstract

The identity and relevance of the ectomycorrhizal (ECM) fungal partners of *Eucalyptus globulus* was investigated in NW Spain, to detect which symbionts mainly support its invasiveness. Root tips of *E. globulus* and of three common native plant species (*Quercus robur*, *Pinus pinaster* and *Halimium lasianthum*) were collected in eucalypt plantations, *Q. robur* forests, *P. pinaster* plantations and shrublands. Fungal taxonomical identity was ascertained by use of rDNA and direct sequencing. We studied diversity, composition and colonization rate of the ECM fungal communities of *E. globulus* to determine if fungal assemblages are host specific (i.e. similar in different habitats) or more dependent on the neighbourhood context. We also identified the type of associations formed (i.e. co-introductions, familiar or novel associations). Twenty-six ECM taxa were associated with *E. globulus*. Most of them engaged in novel associations with eucalypts, whereas only three fungal species were co-introduced Australian aliens. Eucalypt fungal richness, diversity and colonization rate differed between habitats, being higher in native oak forests, whereas in shrublands *E. globulus* showed the lowest colonization rate and diversity. The Australian fungus *Descolea maculata* dominated the eucalypt fungal assemblage and also spread to the native host plants, in all the habitats, posing the risk of further co-invasion.

## Introduction

The movement of alien species around the world is causing major impacts on biodiversity, and forestry is one of the main causes of plant introductions^1,2^. When a species is introduced into a new area, it usually leaves behind its interaction partners, like mutualists (e.g. mycorrhizal fungi and pollinators) and antagonists (e.g. herbivores and pathogens). Its successful establishment in the new area thus highly depends on the formation of biotic interactions with the local community^3–5^. In this regard, the role of antagonists in determining the success of alien species has received the most attention in invasion biology^3^, while the role of mutualisms has been less studied, and especially that of mycorrhizal symbioses^4^. Plant-fungal associations are considered as key drivers of the establishment and success of alien plants^6,7^, but more research is needed to better understand their role as a factor explaining plant invasiveness^8^.

The lack of mycorrhizal symbionts upon arrival to a new area may lead to establishment failure due to mutualist limitation, as occurred in the first attempts with pine plantations in the Southern Hemisphere, before suitable fungi were introduced^9^. Alternatively, mutualist limitation may be avoided by reduced dependence, either inherent or evolved, in the novel range^10^, by co-introductions (when alien symbionts come from the same range of the alien plant), familiar mutualisms (when symbionts that are native from the same area as the alien plant are already present in the new area), or novel associations (when an alien plant or fungus interacts with symbionts not found in its native range)^7^. The dependence, obligate to facultative, of the plant-fungal interaction, its specificity and the availability of compatible mycobionts in the new areas thus become crucial determinants of the type of interactions formed upon arrival^11^. The type of interactions formed will, in turn, have a decisive role on the naturalization, spread and impact of the alien plant^11^. On the other hand, native plant species may serve as hosts of native or alien fungi, which may lead to differences in the availability of compatible fungi among habitats. In case of low host specificity in the plant-fungus interactions, these differences in availability may lead to between-habitat differences in the ECM fungal communities associated with the exotic plant species (context dependency). This situation may lead to differences in the probability of successful establishment and spread of non-native plants depending on the neighbourhood context (i.e. identity of nearby species)^12,13^.

Mycorrhizal fungi are ubiquitous symbionts on plant roots, with arbuscular mycorrhizae (AM) fungi being the most widespread group, and ectomycorrhizal (ECM) fungi the dominant group in temperate forest ecosystems^14,15^. Root colonization by mycorrhizal fungi affects carbohydrate flow, nutrient cycling, increases water uptake, contributes to the aggregation and stabilization of soil particles, and enhances plant tolerance to salinity, herbivory, root pathogens and toxic heavy metals^14^.

*Eucalyptus* (Myrtaceae) is one of the most cultivated plant genera in forestry and has been widely planted around the world, mainly for production of timber and pulp for the paper industry, and as a source of biomass for energy production^16,17^. Among eucalypt species, the Tasmanian blue gum *E. globulus* Labill. (native to South-Eastern Australia, including Tasmania) is the most frequently grown in temperate areas of the world^18^, and has been reported as invasive in southern Europe, North, South and Central America, India, the Pacific Islands, New Zealand and Indian Ocean islands, including Madagascar^19^. In the Iberian Peninsula, its location is mainly limited to the N-NW and SW of Spain (from the Basque Country to Galicia and in Huelva) and to Portugal^20^. Despite the wide distribution of *E. globulus*, our knowledge about its ecology in the new areas of introduction is still poor, especially in regard to its interactions with the local communities, both above- and below-ground (but see for example, Bauhus *et al*.^21^; Calviño-Cancela and Neumann^22^; Calviño-Cancela and Rubido Bará^23^; Becerra *et al*.)^24^.

*Eucalyptus* species form both ECM and AM fungal associations, although ECM fungi are the most important in native forests and plantations^25^. AM fungi are especially important during the early stages of plant development, with a succession from arbuscular mycorrhizae to ectomycorrhizae typically taking place^26^. Being native from the Australasian region, eucalypts have evolved in isolation from the ectomycorrhizal mycobiota associated with *Pinus* and *Quercus* in the Holartic Realm. Considering that the formation of novel associations in new areas may also depend on the degree of phylogenetic relatedness between the alien host plant and the fungal partner^27–29^, the native fungi could have difficulties to associate with such exotic trees under natural conditions. ECM fungal species are in general more host specific than AMF, so frequent host shifts are not expected^14,30^. Experimental works have confirmed that some *Eucalyptus* species perform better with their co-introduced fungi than with locally available mycobionts, emphasising the importance of a long-term coevolution and enhancement of histological and functional compatibility^31,32^. Field studies carried out in areas of introduction (China^32^; Pakistan^33^; Seychelles Islands^34^; England^35^; and Spain^36^) have shown a poor colonization of eucalypt roots by both ecto- and arbuscular mycorrhizae, with a predominance of Australian fungi in some cases. In contrast, the study of Ducousso *et al*.^37^, carried out in Africa and Madagascar, showed rich ECM fungal communities growing in plantations of different *Eucalyptus* species, which were composed of Australian, native African and European fungi. In regard to the Iberian Peninsula, previous studies revealed the presence of Australian fungi in stands of *E. globulus* in NW Spain^38,39^. Successively, up to 12 Australian ECM fungal species were detected in eucalypt stands of SW Spain, which lead Díez^36^ to conclude that native fungi do not form symbioses with eucalypts under natural conditions. In contrast, Lago Álvarez^40^ found a rich ECM fungal community in pure and mixed *Eucalyptus* stands and gardens in NW Spain (90 ectomycorrhizal taxa), including Australian but also native and cosmopolitan fungi. However, that study was based on the identification of sporocarps and may actually reflect the fungal community hosted by a variety of plant species present in eucalypt stands^41^. In any case, while seedling inoculation with compatible ECM fungi was necessary for pine plantations in Africa^42^ and has been recommended for *E. globulus* in China^43^, it has not been necessary for the successful establishment of *E. globulus* in Spain. This suggests that eucalypt seedlings found compatible fungi in the soil.

The aim of the present study is to determine the ECM fungal communities associated with *E. globulus*, in order to better understand the reasons behind the successful establishment of this exotic tree in NW Spain, and its spread in natural habitats outside eucalypt plantations. Our specific aims are: (1) to determine the composition and diversity of the ECM fungal communities associated with *E. globulus* in different habitats in order to test whether these associations are highly host specific or otherwise highly influenced by the neighbourhood context (context dependency); (2) to assess the relative importance of each fungal taxon in the community; and (3) to determine the type of interactions formed (e.g. co-introductions, familiar or novel associations; sensu Dickie *et al*.)^7^, involving both native and alien ECM fungal species. To this end, we sampled the roots of *E. globulus* and of neighbouring plant species in eucalypt plantations and in three habitats dominated by native plant species (*Quercus robur* forests, *Pinus pinaster* plantations and Atlantic shrublands with presence of *Halimium lasianthum*), which are the most common in the region and the most frequently colonized by eucalypts that escape from plantations^23^. We hypothesized that if ECM fungal communities are highly host-specific, we would find the ECM fungal communities associated with *E. globulus* to be similar in all habitats, regardless of the neighbourhood context (i.e. the presence of different ectomycorrhizal host plants). Conversely, if ECM fungal communities are less host-specific but more dependent on the neighbourhood context, we would expect the ECM fungal community of eucalypts to significantly differ between the habitats, showing similarities with the ECM fungal communities associated with the native plant species in each habitat. In this case, we predict the existence of novel associations between *E. globulus* and the native fungi in the three habitats dominated by native species, whereas Australian fungi should still be predominant in the eucalypt plantations.

## Results

A total of 138,762 root tips were examined under the dissecting microscope, of which 34,293 were ECM root tips, initially sorted among 459 morphotypes. The sequence analysis revealed the presence of 31 genera, 62 species and one unknown, black, uncultured ECM fungus (Supplementary Table S2). Nomenclature of fungal taxa follows the Species Fungorum (www.indexfungorum.org). Of the totality of the identified fungi, 24 genera were ECM fungi (23 Basidiomycota and 1 Ascomycota), while the others correspond to non-ECM fungi excluded from the analysis (see Supplementary Table S2).

Irrespective of habitat and site, we found a total of 26 fungal taxa associated with *E. globulus*, 38 taxa with *Q. robur*, 32 taxa were hosted by *P. pinaster* and 11 by *H. lasianthum* (Supplementary Table S2). The unknown, black, uncultured ECM fungus, could be a cryptic species of the *C. geophilum* complex (R. Agerer personal communication), associated with all the studied plants. Of those ECM fungi associated with *E. globulus*, 17 taxa were identified at the species level, whereas eight taxa were identified only at genus level. Among them, 14 species plus two genera that are native in the study region had never been observed, to our knowledge, in the native area of *E. globulus* or have been previously considered non-native in Australia by Vellinga *et al*.^44^, with eight of them never detected in the Australasian region (Supplementary Table S2). We thus assumed that these 16 taxa could not have coevolved with *E. globulus* in the native region and form novel interactions with this tree in the introduced range. In four additional ECM species, their interactions with *E. globulus* have been described as putative novel (Supplementary Table S2), as their status (as native or recently introduced) in the *E. globulus* native range is still uncertain, since they have numerous records in Europe or, in general, in the Northern Hemisphere, and very few and only recent records in Australia, specifically in Victoria and Tasmania, native areas of *E. globulus*. These ECM species may be not native to Australia but have been probably introduced there after European colonization. On the other hand, just three ECM taxa (*Descolea maculata*, *Laccaria lateritia* and *Descomyces* sp.) have been considered as Australian species based on previous studies^44,45^, and have thus been classified as co-introductions (Supplementary Table S2). The rest of the taxa were identified just at genus level and have numerous observations in both Europe and Australia, making it difficult to determine their native region and thus the type of interaction they establish with *E. globulus* (accordingly, the association was referred to as “undetermined” in Supplementary Table S2). A total of 13 taxa are reported here for the first time in association with *E. globulus*, regardless of whether or not they shared their native range with this eucalypt (Supplementary Table S2).

**Figure 1:**
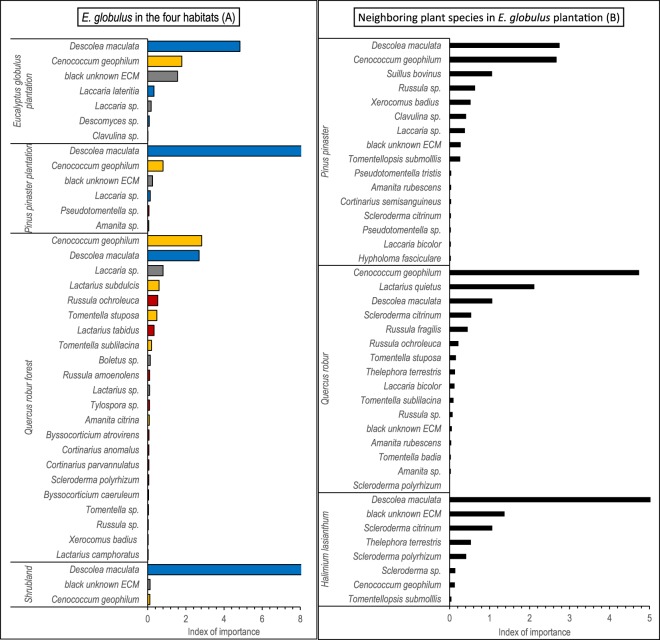
(**A**,**B**) ECM taxa associated with *Eucalyptus globulus* in *E. globulus* plantations, *P. pinaster* plantations, *Q. robur* forests and shrublands (**A**), and with *P. pinaster*, *Q. robur* and *H. lasianthum* in *E. globulus* plantations (**B**). *Descolea maculata*, *Laccaria lateritia* and *Descomyces* sp., represent the only ECM species classified as Australian aliens co-introduced with *Eucalyptus globulus*. The bars represent the sum of relative frequency and relative abundance, which provides an index of the importance of each taxon in the fungal community. Bar colours of Fig. 1A refer to the type of associations between *E. globulus* and the fungal taxa: red for novel associations, yellow for putative novel, blue for co-introductions and grey for undetermined associations (see Supplementary Table S2 for further information).

**Figure 2:**
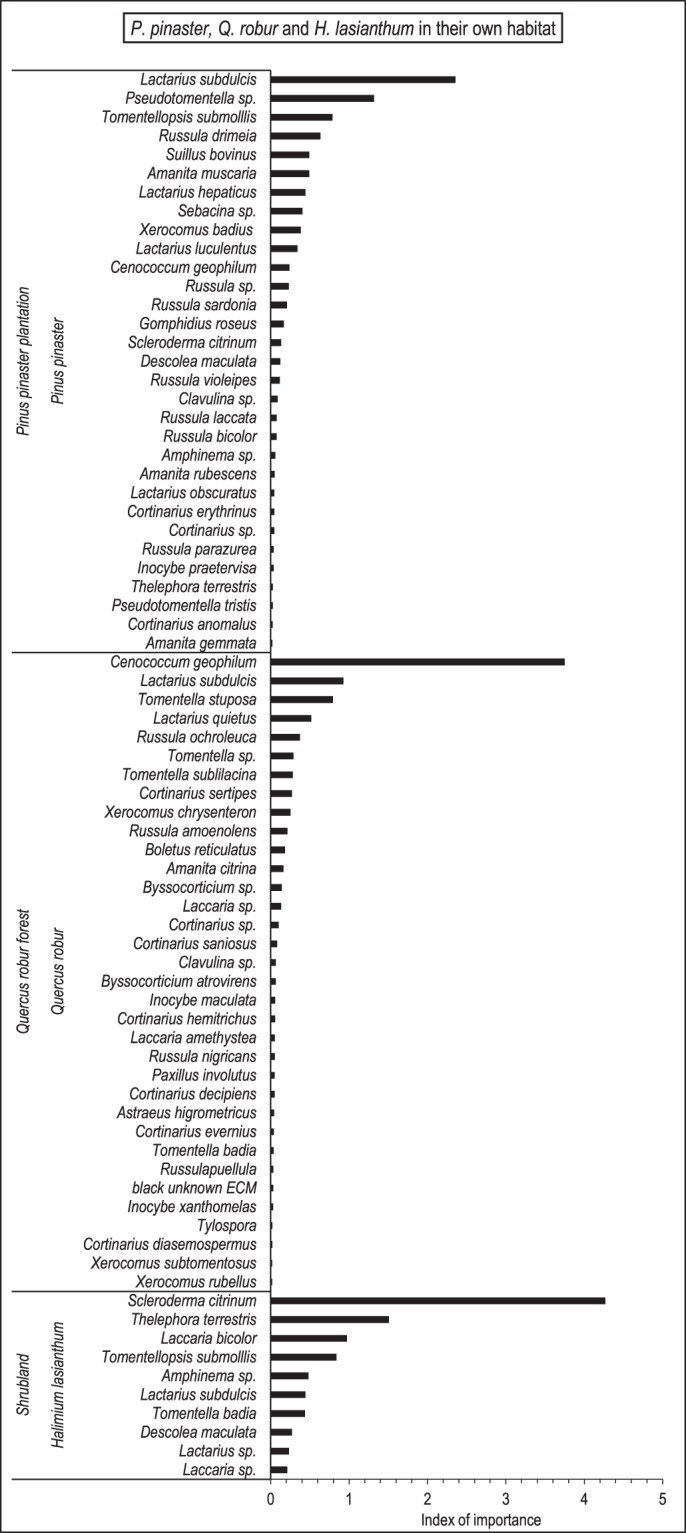
ECM taxa associated with *P. pinaster*, *Q. robur* and *Halimium lasianthum* in their own habitats. *Descolea maculata* was the only ECM species classified as Australian alien co-introduced with *Eucalyptus globulus*, and was found only in pine plantations and shrublands. The bars represent the sum of relative frequency and relative abundance, which provides an index of the importance of each taxon in the fungal community.

Among the Australasian endemics, the presence of *L. lateritia* and *Descomyces* sp. was very infrequent, whereas *D. maculata* was the dominant species in *E. globulus* roots growing in eucalypt plantations, in *P. pinaster* plantations, and in shrublands, sharing this dominant role with *C. geophilum* in *Q. robur* forests (Fig. 1A). *Descolea maculata* is also reported here for the first time associated with all the native host plants studied. This fungus was present not only in eucalypt plantations, where it was the most important ECM in *P. pinaster* (Fig. 1B) and *H. lasianthum* (Fig. 1B), but also in all the other habitats (Fig. 2).

**Figure 3:**
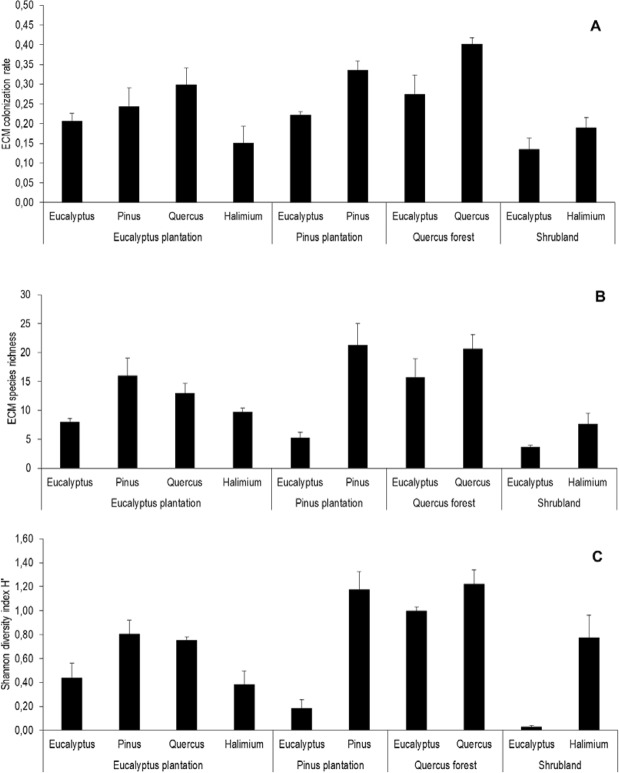
(**A**–**C**) ECM colonization rate (**A**), ECM species richness (**B**), and Shannon diversity index H’ (**C**) of the selected plant species in four habitats. Bars represent the means (with SD) per host plant species in each habitat (three replications per plant species in three localities).

The ECM colonization rate of *E. globulus* varied significantly depending on habitat (F_3,35_ = 7.04, P = 0.001), whereas locality (F_2,35_ = 2.64, P = 0.089) and plant DBH (F_1,35_ = 2.45, P = 0.129) did not influence this variable. For this species, the highest ECM colonization rate was found in *Q. robur* forests (0.27 ± 0.05) and the lowest in shrublands (0.13 ± 0.03) (Fig. 3). In general, *E. globulus* tended to have a lower ECM colonization rate than the other plant species in each habitat, even in eucalypt plantations, where only *H. lasianthum* had a lower colonization rate (Fig. 3).

Fungal species richness of *E. globulus* was affected by habitat (F_3,35_ = 27.66, P < 0.001), but not by locality (F_2,35_ = 0.67, P = 0.520) or plant DBH (F_1,35_ = 6.93, P = 0.066). *Eucalyptus globulus* showed a significant increase of fungal species when growing in *Q. robur* forests (15.67 ± 3.28) compared to eucalypt plantations (8 ± 0.58), where it showed a similar fungal richness than in shrublands (3.67 ± 0.33) or pine plantations (5.33 ± 0.88) (Fig. 3). Similarly, Shannon diversity index (H’) significantly differed depending on habitat (F_3,35_ = 22.95, P < 0.001), with no effect of locality (F_2,35_ = 0.19, P = 0.831) or plant DBH (F_1,35_ = 0.04, P = 0.840). In this case, the highest diversity was found in *Q. robur* forests (1.00 ± 0.03) and the lowest in shrublands (0.03 ± 0.19) (Fig. 3).

For *P. pinaster*, the ECM colonization rate was significantly affected by habitat (F_1,19_ = 6.09, P = 0.026), but not by locality (F_2,19_ = 0.47, P = 0.706) or plant DBH (F_1,19_ = 0.50, P = 0.492). In particular, the ECM colonization rate of pines growing in eucalypt plantations was 25% lower than in the native habitat.

In contrast, fungal species richness of *P. pinaster* did not significantly change depending on habitat (F_1,19_ = 2.09, P = 0.169), nor for locality (F_2,19_ = 0.02, P = 0.984) or plant DBH (F_1,19_ = 0.95, P = 0.346). Shannon diversity index (H’) was affected by habitat (F_1,19_ = 4.98, P = 0.043), being higher in pine plantations (Fig. 3), but not by locality (F_2,19_ = 1.24, P = 0.319) or plant DBH (F_1,19_ = 0.76, P = 0.397).

*Quercus robur* growing in eucalypt plantations had a significantly lower ECM colonization rate than in its native habitat (F_1,17_ = 5.20, P = 0.040) (Fig. 3), whereas locality (F_2,17_ = 0.60, P = 0.563) and plant DBH (F_1,17_ = 1.04, P = 0.326) had no effect. Similarly, fungal species richness associated with this plant was higher in oak forests (F_1,17_ = 10.0, P = 0.007) (Fig. 3). Locality (F_2,17_ = 0.99, P = 0.399) and plant DBH (F_1,17_ = 0.47, P = 0.504) did not influenced this variable. Shannon diversity index (H’) was higher in native forests (F_1,17_ = 8.39, P = 0.012), but locality (F_2,17_ = 0.17, P = 0.848) and plant DBH (F_1,17_ = 0.01, P = 0.923) had no effect.

ECM colonization rate of *Halimium lasianthum* was neither affected by habitat (F_1,18_ = 0.90, P = 0.357), nor by locality (F_2,18_ = 0.36, P = 0.706). Similarly, fungal species richness did not change with habitat (F_1,18_ = 0.00, P = 1) or locality (F_2,18_ = 3.08, P = 0.076). Finally, Shannon diversity index (H’) did not change between shrubland and eucalypt plantation (F_1,18_ = 3.25, P = 0.092) or with locality (F_2,18_ = 2.10, P = 0.157).

The ratio between the observed and the estimated species richness (using the Chao2 estimator) ranged between 41.9% and 100%, with the lower ratios corresponding to *Q. robur* in eucalypt plantations, *E. globulus* in *Q. robur* forests and *P. pinaster* in pine plantations, which suggests some undersampling of species in these cases (Supplementary Fig. S1 and Table S3).

The ECM taxa composition was significantly affected by host plant species within habitats (PERMANOVA analysis; Supplementary Table S4). However, the effects of locality and its interaction with the host plant species within each habitat were not significant. There were differences between habitats for a given plant species: the ECM communities associated with *E. globulus* were significantly different between eucalypt plantations and oak forests, but similar between shrublands, pine and eucalypt plantations. The ECM communities associated with *P. pinaster* and *H. lasianthum* differed also when comparing eucalypt plantations and their own habitats (pine plantations and shrublands, respectively), while the communities associated with *Q. robur* were similar in oak forests and eucalypt plantations. There were also differences in ECM fungal community composition between host plant species within a given habitat (Supplementary Table S5). Focusing on *E. globulus*, there were significant differences with *Q. robur* in oak forests, with *P. pinaster* in pine plantations and with *H. lasianthum* in shrublands, as well as between *E. globulus* and *Q. robur* in eucalypt plantations, but not with *P. pinaster* and *H. lasianthum* in eucalypt plantations.

## Discussion

Four strategies allow plants to spread without being limited by the lack of mutualists: not being dependent on mutualists, to form familiar associations, to form novel mutualisms, or to co-invade with its own mutualists from the native area^7,46^. Novel mutualisms were the most frequent association motif in our study, whereas only three species were classified as co-introduced Australian aliens. This shows the low eucalypt-fungal symbiont specificity, with host plant shifting to native ECM fungi seeming an important mechanism to avoid mutualism limitation in the introduced range (see also Jairus *et al*.^47^ and Ducousso *et al*.^37^ in Africa). Despite the numerous associations with native fungi, the quantitatively most important association was engaged with the Australian *D. maculata*, which suggests a key role in the establishment success of *E. globulus*. The genus *Descolea* has about 15 described species, which may be associated with members of the family Pinaceae, Fagaceae and Myrtaceae^39^. Possibly, it was unintentionally introduced with eucalypt roots or soil, as it generally occurs worldwide with other exotic fungal species^44^. It is a generalist species, and its high abundance and frequency in our study sites indicates the compatibility not only with native host species but also with the local climatic and edaphic characteristics. This exotic symbiont dominated the ECM community associated with *E. globulus* in all the habitats and established associations with all the plant species studied, being a preponderant symbiont of *P. pinaster* and *H. lasianthum* in eucalypt plantations. Its ubiquitous presence in the study area points to a co-invasion of this fungus with *E. globulus*. Interestingly, this ECM species was never found in association with *Q. robur* in oak forests, despite its compatibility with *Q. robur* in eucalypt plantations. This suggests that a highly diverse ECM community may provide some resistance to competitive displacement by an exotic species^46^, or a higher affinity of *Q. robur* with other species (e.g*. C. geophilum)*.

It has been suggested that introduced ECM fungi in forestry habitats may persist during a short period in the field, being outcompeted by native species^48^. This is not the case of *D. maculata*, whose presence in Spain was detected at the beginning of the 1990s in *E. globulus* plantations in NW Spain^39^. Its ability to associate with all the native species studied has probably played a key role in securing its persistence in the introduced area. The association between *D. maculata* and *E. globulus* seems a case of subsidized mutualism (sensu Dickie *et al*.)^7^ in which native plants facilitate the success of the exotic fungi, favouring the increase of its local populations. This would increase inoculum availability, favouring the mutualism with the exotic *E. globulus*, ultimately facilitating the penetration of *E. globulus* on native habitats outside plantations. Further investigations are needed on the functional role of *D. maculata* in its symbiotic association with *E. globulus* and the traits that may make it an ideal co-invasive ECM species.

The ascomycete *C. geophilum*, a native species, also has an important role as symbiont of *E. globulus* in the study area. This species is one of the most common and globally abundant ECM fungus, distributed from boreal to temperate regions^49^, but its presence in the Southern Hemisphere and in Australia in particular seems to be recent and for this reason we have classified its association with *E. globulus* as putative novel (see Supplementary Table S2). It shares a dominant role with *D. maculata* in *Q. robur* forests and is present in association with *E. globulus* in all the other habitats studied. The dominance of *C. geophilum* in the ECM community of *Q. robur* forests shows the ability of this fungus to shape the ECM assemblage also in a rich and complex habitat (see for example Trocha *et al*.^50^ for the *Q. rubra* fungal community). The association between C. *geophilum* and *E. globulus* seems also a case of subsidized mutualism, in this case involving a native fungus^7^. The main symbionts in association with *E. globulus* are thus fungi with broad host ranges.

Both the host plant species and the habitat were important determinants of the ECM community, in terms of diversity as well as in colonization rate. We found that, in a given habitat, different plants tend to host different ECM communities, which highlights a certain degree of specificity in mycorrhizal interactions. But there was also a clear influence of neighbourhood context, so that the same plant species tended to host different ECM communities in different habitats. Focusing on *E. globulus*, it hosted less ECM taxa and with lower colonization rate than the native species in all habitats, even in eucalypt plantations. This might be related to a certain degree of incompatibility with the available fungi assemblage. Surprisingly, it was in native oak forests where *E. globulus* showed the richest ECM communities with the highest colonization rates, which points to the subsidizing role of native host species^7^. *Eucalyptus globulus* can indeed establish interactions with native fungi, but, of them, only *C. geophilum* showed a high colonization rate, while the rest of native fungi had low importance. These fungal mutualists could thus have difficulties to sustain a healthy mycelium network with *E. globulus* as host^41^, leading to the impoverishing of ECM fungal communities associated with this species.

Generally, the ECM community was more diverse and the intensity of the fungal association (as estimated by colonization rate) was higher in native oak forests than in plantations or in shrublands. In oak forests, the high diversity of ECM fungi associated with *Q. robur* together with a high diversity of other potential host species in the understory^41^ probably increases the diversity of the fungal inoculum available, which enhances the opportunity of compatible associations with eucalypt roots.

Despite the seemingly facilitating effect of oaks, subsidizing the formation of mutualistic fungal interactions with high diversity and colonization rate, native oak forests have shown more resistance to the establishment of *E. globulus* seedlings than pine plantations or shrublands^23^. This might be due to other factors (such as litter or shade, see Calviño-Cancela *et al*.^51^) having more importance in determining seedling establishment than mycorrhizal interactions. Also, a high diversity and colonization rate of eucalypts in oak forests do not necessarily translate into higher tree performance. It may depend more on the identity of mutualistic partners, with the quality of the nutrient trade-off being more important than the quantity in determining mutualist effectiveness^52^. In oak forests, *E. globulus* was indeed associated with more fungal species, but precisely in this habitat *D. maculata* was not dominant. If *D. maculata* was more effective in favouring *E. globulus* performance, their lower dominance in eucalypt roots in this habitat could result in lower colonization ability of *E. globulus*. Further studies are needed to determine the differential performance of *E. globulus* depending on its associated mutualist fungi.

The ECM diversity found in eucalypts growing in oak forests contrasts with the lower diversity found in pine plantations, despite the high number of ECM species associated with the dominant *P. pinaster* in this habitat, (31 ECM taxa), which was similar to that exhibited by *Q. robur* in oak forests (34 ECM taxa). Possibly, the high phylogenetic distance between *Eucalyptus* (Angiosperm) and *Pinus* (Gymnosperm) may account for the difficulty of the fungal host shift, as it has been shown for pathogens^28,53^, and for ECM symbionts^29^. Also, the role of some physical and chemical soil factors that may hinder the compatibility between ECM species and *E. globulus* roots cannot be discarded^54^. Despite this low diversity, *E. globulus* roots attained a relatively high colonization rate, dominated by *D. maculata*.

However, it was in shrublands where *E. globulus* showed the lowest diversity of ECM taxa. This may be due to the dominance of ericaceous and leguminous species in this habitat^41^, which do not form ECM and consequently do not provide the soil with fungal propagules. *Halimium lasianthum* does form ECM associations, but it is not a dominant species in this habitat. Moreover, the ECM community associated with *H. lasianthum* is relatively poor, as compared to that of the other native species studied (*P. pinaster* and *Q. robur*), all this contributing to limit the availability of ECM fungal inoculum, both in quantity and diversity.

The context dependency of the *E. globulus* ECM community possibly explains why studies developed exclusively in eucalypt plantations have indicated the existence of a low fungal diversity^32,33,35,36^. However, the study carried out by Díez^55^ in a natural forest dominated by *Q. pubescens*, *Q. ilex* and *Q. faginea* in Central Spain, with patches of *E. camaldulensis* and *E. globulus*, found low diversity of ECM fungi, with preponderance of Australian species in eucalypts, and no host shifts by exotic fungal species toward native trees. This is in clear contrast with the high number of novel associations of *E. globulus* with native fungi found in the present study. Possibly, different sampling methods, climatic conditions and soil features account for it.

In conclusion, we have shown that the spread of Australian fungal taxa and the establishment of novel interactions enhanced the mycorrhization of eucalypts in NW Spain. This made inoculation of the seedlings with compatible ECM fungi unnecessary and facilitated the spread of *E. globulus* in natural habitats, outside eucalypts plantations. Context dependency strongly shaped the ECM community of *E. globulus*, with marked between-habitat differences in ECM species diversity, composition and colonization rate. Mycorrhization was enhanced in native oak forests, where ECM species diversity and abundance were greater. The Australian fungus *D. maculata* was widespread in the habitats and was associated with all the studied plant species, which is a cause of concern. Intermingling root systems of the exotic and native host trees allows host shifting of ECM fungi and facilitate the risk of co-invasion by the exotic *D. maculata*. Our results highlight the importance of investigating the spread of exotic species across multiple ecological settings in order to better understand the reasons behind successful establishment.

## Materials and Methods

### Study area

The study was carried out in Galicia (NW Spain), which is the most important forestry region of Spain. It is characterized by an Atlantic humid climate without large frost periods and with precipitation all year round^56^. The average annual temperature varies between 8 and 14 °C. Annual rainfall generally ranges between 600 and 2500 mm, with some sites over 3000 mm per year. The present research was conducted at Pontevedra province, and was replicated in three localities: Cangas (42°17′N/8°46′W, altitude 200–350 m), Pontecaldelas (42°20′N/8°33′W, altitude 30–250 m) and Covelo (42°14′N/8°21′W, altitude 350–550 m), at a distance between 20–35 km from one another. Localities were selected so that the four study habitats were present at a short distance to one another (<3 km). In each locality, we selected an *E. globulus* plantation, a native *Q. robur* forest, a *P. pinaster* plantation and a shrubland patch. Shrublands and *Q. robur* forests have the most diverse plant communities, while *E. globulus* plantations tend to be the poorest^41^.

### Soil characterization

Soils are typically acidic and sandy, contain a high proportion of coarse fragments, and have a high content of organic matter and low levels of nutrients^57^. We analysed the soil parameters in each of the studied habitats in the three localities, with 3 samples per site (a total of 36 samples). We collected soil samples of 20 × 20 cm and 20 cm depth. Soil pH, organic matter (%), organic C, available soil P, K, Mg, N, C/N ratio, Ca, K, Mg, Na, Al cations, Cation Exchange Capacity (CEC), Ca/Mg and K/Mg ratios were determined. Soil analyses were carried out at the Estación Fitopatológica de Areeiro (http://www.efa-dip.org). Results are summarized in Supplementary Table S1.

### Experimental design and root sampling procedure

We sampled in eucalypt plantations the roots of *E. globulus* and of other three mycorrhizal plants (*Q. robur*, *P. pinaster* and *H. lasianthum*). Also, in three habitats dominated by native plants (i.e. *Quercus robur* forests, *Pinus pinaster* plantations and Atlantic shrublands), we sampled the roots of the most predominant plant species (i.e. *Q. robur* in *Q. robur* forests, *P. pinaster* in *P. pinaster* plantations, and *H. lasianthum* in Atlantic shrublands) and the roots of invasive *E. globulus* trees. Although *H. lasianthum* was not the most common species in Atlantic shrublands, with this habitat being mainly dominated by non-ectomycorrhizal species, it was the most common among those forming ECM. Three plant individuals per species and per habitat were randomly selected and sampled in each locality, making a total of 90 root samples (30 samples/locality). Diameter at breast height (DBH) was measured for each tree sampled, in order to control for the influence of tree size on the fungal community. To avoid spatial autocorrelation (similarity) of the ECM community, the distance between sampled plants was greater than 4 m^58^.

Approximatively 200 cm of fine roots were collected for each plant. The main roots of each selected tree were unearthed and tracked until they formed fine roots. This procedure enabled us to verify the physical links between ECM root tips and the host plant, avoiding the inclusion of roots belonging to non-target plants. For the shrub *H. lasianthum*, we dig up the whole ramet with the root system. Fine roots were introduced in marked plastic bags, transported to the laboratory, and stored at 4 °C. To minimize storage time, we collected in the field only 3–4 samples on average in each sampling date (a total of 24 samplings were carried, from September 2014 to May 2016). Samples were temporally interspersed between host species, habitats and localities in order to minimize possible biases due to seasonal variation. Following Agerer^59^, the mycorrhizal root tips were sorted into different morphotypes based on colour, shape, texture, ramification type, occurrence of cystidia, mantle type, emanating hyphae and rhizomorphs. For each sampled plant, the morphotypes were placed individually in a 1.5-ml Eppendorf tube and stored at −20 °C.

### Molecular analysis

The taxonomic identity of the ECM was ascertained by PCR amplification and direct sequencing of the Internal Transcribed Spacer (ITS)^60^. Fungal DNA was extracted from fresh ECM root tips using the EZNA Fungal DNA Kit (Omega Bio-Tek, USA), and successively purified with the Power Clean® Pro DNA Clean up Kit (MoBio Laboratories Inc.) according to the manufacturer’s instructions. DNA samples were run by gel electrophoresis, with 1% agarose gel and 3 µl of ethidium bromide, at 120 V for 20 min. Results were visualized under a UV-transilluminator BioDoc-it LCD/FI-26 (UVP Cambridge). A gross estimation of DNA concentration and molecular length was obtained by using GeneRuler 100 bp DNA Ladder (Thermo Scientific^TM^). PCRs were conducted in 30 µl volume reaction containing MgCl_2_ 50 mM (BIORAD), DreamTaq Green PCR Master Mix (2X) (Thermo Scientific), Bovine Serum Albumin (BSA) 10 × (Thermo Scientific), pure water nuclease-free (Thermo Scientific) and 1 µl of undiluted DNA (concentration ranged between 50 and 100 ng/µl). The universal primers ITS1, ITS4, ITS1F, ITS4B, ITS5, and ITS4A commonly used in ECM community studies^61^ were used to amplify the ITS1, 5.8 S and ITS2 regions of the nuclear rDNA. The amplification conditions were optimized for each primer combination, but the general reaction protocol was as follows: 94 °C for 3 min, followed by 40 cycles of 94 °C for 30 s, 55 °C for 30 s, 72 °C for 1 min and a final extension step of 72 °C for 10 min. PCR reaction products were electrophoresed in a 2% agarose gel.

Part of the samples was amplified by using the direct PCR procedure^62^, which is faster and less expensive than conventional methods, and allows to amplify ITS fragments directly from the root tips, without performing the DNA extraction and purification. For this method, the PCR reaction protocol was: 95 °C for 6 min, followed by 30 cycles of 94 °C for 30 s, 55 °C for 30 s, and 72 °C for 1 min and a final extension step of 72 °C for 10 min.

PCR products were purified and sequenced by Macrogen Laboratories (http://www.macrogen.com). Forward and reverse sequences were aligned and edited using BioEdit Sequence Alignment Editor 7.2.6 (http://www.mbio.ncsu.edu/bioedit/bioedit.html). All sequences were identified to genus or species level by querying the GenBank database, using the nucleotide– nucleotide (blastn) BLAST search option, available through the National Center for Biotechnology Information (NCBI). We considered only sequences with 80–100% query coverage and E value (expectation value) equal to zero. Assignment to taxonomic categories was performed using the following criteria: sequence similarity between 90 and 97% led to identification at the genus level, whereas a value ≥ 98% gave a match for species identification. ECM root tips formed by the ascomycete *Cenococcum geophilum* were sequenced just once and successively designated without sequencing thanks to their unique morphological characteristics.

### Geographical distribution and association motif

We have based our classification of plant-fungus associations motif (i.e. novel, putative novel and co-introduced) on the information available in the literature about the native and alien ranges of the fungal taxa recorded (see for example the revision by Vellinga *et al*.^44^), and on the distribution maps and records provided at www.discoverlife.org, http://nzfungi.landcareresearch.co.nz/html/search_index.asp, http://www.ala.org.au/blogs-news/fungimap-putting-australian-fungi-on-the-map/, and www.gbif.org. Caution should be taken, however, given the uncertainty due to limited historical records in some continents and possible biases, which means that inconspicuous species may pass unnoticed for long or that some species are first described, or even known only, from their introduced range^7,63^.

### Colonization rate and diversity

ECM colonization rate (total ECM root tips/total root tips per plant) was calculated by using the gridline intersect method under a dissecting microscope^64^. Two replicates of 50 pieces (approximately 1 cm) of roots were examined per sampled plant. The fragments were randomly dispersed in a 9-cm-diameter Petri dish with grid lines. Once the taxonomic identity of the ECM root tips was obtained, after molecular verification, the relative abundance (total root tips colonized by each taxon/total ECM root tips per sampled plant), the absolute frequency (number of plants with presence of a taxon/total sampled plants) and the relative frequency (absolute frequency of a taxon/$\sum$ absolute frequency of all taxa) of each fungal taxon per host plant species and per habitat were calculated in order to assess the importance of each fungal taxa in the community. Following Brundrett *et al*.^64^, we used the sum of relative frequency and relative abundance as an index of the importance of each taxon in the fungal community. We computed the species richness (S) of ECM, and Shannon–Wiener diversity index (H’).

### Statistical analyses

Prior the analyses, variables were checked for normality by Kolmogorov–Smirnov test and Bartlett’s test for homogeneity of variances. Data expressed as ratios were previously subjected to logarithm transformation. Significant variation of soil parameters between the habitats was investigated with a one-way-ANOVA. The existence of significant differences of the ECM colonization rate, species richness and diversity (H’) were assessed by ANOVA, using host plant species within habitat as fixed factor with 10 levels (corresponding to the 10 combinations between plant species and habitats that we sampled: *E. globulus* in eucalypt plantation, *E. globulus* in pine plantation, *E. globulus* in shrubland, *E. globulus* in oak forest, *P. pinaster* in pine plantation, *P. pinaster* in eucalypt plantation, and so on), locality as random factor (3 levels) and DBH (only for the tree species*: E. globulus*, *P. pinaster* and *Q. robur*) as covariate. Pairwise comparisons were done with LSD (Least Significant Difference) Fisher’s test. The analyses were performed with GenStat release 10.2 (VSN International, Hemel Hempstead, UK).

Species accumulation curves with 95% confidence intervals and 500 permutations were computed to assess the efficiency of ECM sampling for each habitat and host plant species, and were computed with EstimateS^65^. To estimate the true species richness we used Chao2 estimators^66^.

To test for differences in species composition and to estimate the components of variation we used the permutational multivariate analysis of variance (PERMANOVA), with the number of root tips of each mycorrhizal taxon as the variates. We used host species within habitat as a fixed factor with 10 levels and locality (3 levels) as a random factor, and their interactions. PERMANOVA analyses were based on Bray-Curtis similarities. We used PRIMER 7.0.13^67^ with the PERMANOVA + 1 add-on^68^ for these analyses. The analysis was performed using 9999 permutations, with permutation of residuals under a reduced model, fixed effects sum set to zero and type III sums of squares. Significance was declared at P < 0.05. Means are reported with the standard deviation (mean ± SD).

## Supplementary information

Supplementary Informations
