# Supplementary material for: Context dependency, co-introductions, novel mutualisms, and host shifts shaped the ectomycorrhizal fungal communities of the alien tree Eucalyptus globulus
Source: Sci Rep. 2019 May 9;9:7121. doi: 10.1038/s41598-019-42550-x (PMC6509251; doi:10.1038/s41598-019-42550-x)
Supplement: Supplementary file 1 — Supplementary Informations [file 41598_2019_42550_MOESM1_ESM.pdf]

Authors: Serena Santolamazza-Carbone, Mónica Durán-Otero, María Calviño Cancela

Title: Context dependency, co-introductions, novel mutualisms, and host shifts shaped the ectomycorrhizal fungal communities of the alien tree *Eucalyptus globulus*

Journal: Scientific Reports

Supplementary Table S1. Soil parameters (mean ± SD) of the four habitats after pooling data from the three experimental sites. Three soil samples per locality and habitat were collected. CEC = Cation Exchange Capacity. P-values = results of one-way ANOVA of difference between habitats. Significance was declared at P < 0.05. Letters in bold show the existence of a significant difference. LSD = Fisher's test Least Significant Difference.

	***Eucalyptus* plantation**	***Pinus* plantation**	***Quercus* forest**	**Shrubland**	**P-values**	**LSD**
**pH (H_2O)**	4.47±0.05	4.56±0.04	4.60±0.11	4.64±0.17	0.681	0.343
**Organic matter (%)**	14.31±2.92	15.67±0.48	17.08±1.97	10.17±2.42	0.204	7.01
**Organic C (%)**	8.30±1.69	9.09±0.28	9.91±1.15	5.90±1.40	0.204	4.068
**Available P (ppm)**	9.56±0.40	7.78±2.15	12.22±3.72	6.78±0.78	0.375	7.15
**Available K (ppm)**	80±9.17 **b**	63.70±11.41 **b**	134±15.10 **a**	46.22±9.77 **b**	0.004	37.81
**Available Mg (ppm)**	37.33±8.72	33.56±15.48	78.22±26.61	17.78±5.79	0.132	53.02
**N (%)**	0.48±0.15	0.52±0.06	0.63±0.09	0.35±0.08	0.341	0.327
**C/N**	18±1.53	18.67±1.33	16±0.58	16.33±1.33	0.416	4.067
**Ca (cmol/kg)**	0.12±0.02 **c**	0.20±0.08 **b**	0.58±0.12 **a**	0.24±0.12 **b**	0.038	0.306
**K (cmol/kg)**	0.20±0.02 **b**	0.16±0.03 **b**	0.34±0.04 **a**	0.12±0.02 **b**	0.004	0.095
**Mg (cmol/kg)**	0.30±0.07	0.27±0.12	0.63±0.21	0.14±0.05	0.132	0.424
**Na (cmol/kg)**	0.18±0.03	0.17±0.01	0.17±0.03	0.07±0.03	0.076	0.088
**Al (cmol/kg)**	6.18±0.79	5.77±0.29	6.03±0.42	3.90±0.81	0.099	2.018
**CEC**	6.98±0.92	6.58±0.14	7.74±0.69	4.47±0.99	0.076	2.491
**Ca/Mg**	0.33±0.19 **b**	0.74±0.21 **b**	1±0.00 **a**	1.67±0.38 **a**	0.024	0.778
**K/Mg**	0.77±0.10	0.92±0.22	0.65±0.18	0.95±0.15	0.585	0.555

Supplementary Table S2. Ectomycorrhizal fungal species found in association with *Eucalyptus globulus* (E), *Pinus pinaster* (P), *Quercus robur* (Q), and *Halimium lasianthum* (H), in four habitats (*E. globulus* plantation = Ep; *P. pinaster* plantation = Pp; *Q. robur* forest = Qf; and shrubland = S). Taxonomical identity was ascertained by direct sequencing of the ITS regions and BLAST search. Names in bold indicates that this is the first time that the taxon is reported in association with *E. globulus*. Geographical distribution have been assessed by using the available literature (see for example Vellinga et al. 2009) and the records and global mapper provided by the on-line encyclopedia of life www.discoverlife.org, the catalogue of New Zealand Fungi (http://nzfungi.landcareresearch.co.nz/html/search_index.asp), the Atlas of Living Australia website (http://www.ala.org.au/blogs-news/fungimap-putting-australian-fungi-on-the-map/), and the Global Biodiversity Information Facility web (www.gbif.org). Abbreviations in geographical distributions read as follows: Nea = Neartic region (North America, Greenland, Central Florida, highland of Mexico); Neo = Neotropical region (South Florida, lowlands of Mexico, Central America, South America, Caribbean islands); Pal = Paleartic region (Europe, North Africa, North Asia, northern and central parts of the Arabian peninsula); Afr = Afrotropical region (Africa South of the Sahara, Madagascar and the southwestern area of the Arabian peninsula); Hi = Hindomalayan region (South and Southeast Asia and southern parts of East Asia); Aus = Australasian region (Australia, New Guinea, New Zealand, New Caledonia, Tasmania, Pacific island groups). Aus* = taxon present in the Australasian region but not in the native range of *E. globulus*.

Association motif refers only to those species associated to *Eucalyptus globulus* according to Dickie et al. (2017). The term novel indicates the association between *E. globulus* and those ECM species which are not spread in the native range of this eucalypt (i.e. South-Eastern Australia, including Tasmania). Novel/familiar refers to the association with ECM species native in NW Spain, which status (as native or recently introduced) in the *E. globulus* native range is still uncertain because they have very few and recent records. The term co-introduction refers to those ECM species native in the Australian continent but alien in Europe, which were presumably co-introduced in NW Spain with *E. globulus*.

Non-ECM fungi were also identified, but were excluded from further analyses. They were the litter decomposers *Mycena zephirus* (Basidiomycota, Mycenaceae) and *Hypholoma fasciculare* (Basidiomycota, Strophariaceae), the ericoid mycorrhizae *Meliniomyces bicolor* (Ascomycota, Leotiomycetes) and *Rhyzoscyphus* sp. (Ascomycota, Leotiomycetes), the saprobic *Coltricia* sp. (Basidiomycota, Hymenochaetaceae), the soil fungus *Trichosporon porosum* (Basidiomycota, Trichosporonaceae), and the opportunistic, non-mycorrhizal fungus *Xenasmatella* sp. (Basidiomycota, Xenasmataceae). Also, some uncultured Helotiales (Ascomycota), reported as secondary colonists of ECM root tips (Tedersoo et al. 2009), were found in *P. pinaster* and *E. globulus* samples. The ericoids *M. bicolor* and *Rhyzoscyphus* have been found in a *Q. robur* forest patch, coming from *Q. robur* and *E. globulus* roots. Recently, ericoid mycorrhizal fungi have been described in association with *Q. ilex*, however, the possibility that some sharing may occur between ericaceous plants and ECM trees is still under debate (Bergero et al. 2000).

**Identification (Blast search)**	**Similarity (%)**	**Size (bp)**	**Habitat, host plant**	**Genbank accession**	**Geographic distribution**	**Association motif with *E. globulus***
Amanita citrina (JN235140)	98	857	Qf, Q<br>Qf, E	KY587527	Nea, Neo, Pal, Afr, Hi, Aus	Putative novel
Amanita muscaria (JF899546)	99	588	Pp, P	KY587526	Nea, Neo, Pal, Hi, Aus	Not associated with *E. globulus*
Amanita sp. (*A. gemmata* HQ604824)[1]	94	760	Ep, P<br>Pp, E<br>Pp, P	KY596001	Nea, Neo, Pal, Afr, Hi, Aus*	Novel[2]
Amanita rubescens (AJ889923)	99	789	Ep, Q<br>Ep, P<br>Pp, P	KY595996	Nea, Pal, Aus	Not associated with *E. globulus*
Amphinema sp. (JN943925)	96	694	S, H	KY595998	Pal, Nea, Hi, Aus	Not associated with *E. globulus*
Astraeus hygrometricus (FJ710188)	99	818	Qf, Q	KY596000	Nea, Neo, Pal, Afr, Hi, Aus	Not associated with *E. globulus*
Uncultured ECM fungus (FR731296)	99	608	Ep, Q<br>Ep, E<br>Ep, H<br>Ep, P<br>Pp, E<br>Qf, Q<br>S, E	KY609918		Undetermined
Byssocorticium sp. (JQ888156)	95	609	Ep, Q<br>Qf, Q	KY611395	Pal	Not associated with *E. globulus*
Byssocorticium atrovirens (AJ889936)	99	729	Qf, Q<br>Qf, E	KY595993	Nea, Pal	Novel
Byssocorticium caeruleum (GQ162814)	99	697	Qf, E	KY595997	Pal	Novel
Boletus sp. (FN669289)	100	778	Qf, E	KY595994	Pal, Nea, Neo, Afr, Hi, Aus	Undetermined[3]
Boletus reticulatus (KC422595)	99	856	Qf, Q	KY595992	Pal, Nea, Hi	Not associated with *E. globulus*
Cenococcum geophilum (KC967407)	99	496	Qf, Q<br>Qf, E<br>Ep, E<br>Ep, P<br>Ep, Q<br>Ep, H<br>Pp, P<br>Pp, E<br>S, E	KY595999	Nea, Neo, Pal, Afr, Hi, Aus*	Putative novel[4]
Clavulina sp. (KM576341)	97	758	EP, E<br>Qf, Q<br>Pp, P	KY611396	Nea, Neo, Pal, Afr, Hi, Aus	Undetermined[3]
Cortinarius anomalus (AY669645)	99	492	Qf, E<br>Pp, P	KY595995	Nea, Neo, Pal, Hi, Aus*	Novel[5]
Cortinarius decipiens (AJ889946)	99	675	Qf, Q	KY640616	Nea, Pal, Aus	Not associated with *E. globulus*
Cortinarius diasemospermus (AJ889970)	99	689	Qf, Q	KY640619	Pal	Not associated with *E. globulus*

Cortinarius erythrinus (AY669690)	98	706	Pp, P	KY659394	Pal, Nea, Hi	Not associated with *E. globulus*
Cortinarius evernius (HQ604723)	98	742	Qf, Q	KY640618	Nea, Pal, Aus	Not associated with *E. globulus*
Cortinarius hemitrichus (DQ0977870)	99	640	Qf, Q	KY654987	Nea, Pal, Aus	Not associated with *E. globulus*
Cortinarius parvannulatus (AY669664)	98	707	Qf, E	KY640615	Pal, Nea	Novel
Cortinarius saniosus (DQ102672)	98	760	Qf, Q	KY654986	Nea, Pal, Aus	Not associated with *E. globulus*
Cortinarius semisanguineus (FJ039598)	99	750	Ep, P	KY654955	Nea, Neo, Pal, Hi, Aus	Not associated with *E. globulus*
Cortinarius sertipes (AJ889969)	99	725	Qf, Q	KY640617	Nea, Pal	Not associated with *E. globulus*
Descolea maculata (DQ192181)	99	770	Qf, E<br>Ep, E<br>Ep, P<br>Ep, Q<br>Ep, H<br>Pp, P<br>Pp, E<br>S, E<br>S, H	KY654754	Pal, Aus	Co-introduced
Descomyces sp. (JF714650)	90	862	Ep, E	KY654985	Pal, Neo, Aus	Co-introduced
Gomphidius roseus (DQ534570)	100	636	Pp, P	KY971616	Pal, Hi	Not associated with *E. globulus*
Inocybe sp.<br>(*Inocybe maculata* AM882958)	89	773	Qf, Q	KY655007	Pal. Nea, Hi	Not associated with *E. globulus*
Inocybe praetervisa (AM882720)	99	734	Pp, P	KY655012	Pal. Nea, Hi	Not associated with *E. globulus*
Inocybe xanthomelas (FN550895)	98	800	Qf, Q	KY655005	Pal. Nea, Hi	Not associated with *E. globulus*
Laccaria sp. (JX030275)	96	738	Qf, Q<br>Qf, E<br>Ep, E<br>Ep, P<br>Ep, Q<br>Pp, P<br>Pp, E<br>S, E<br>S, H	KY655006	Pal, Nea, Neo, Afr, Hi Aus	Undetermined[3]
Laccaria amethystea (AF539737)	99	818	Qf, Q	KY655004	Pal, Nea, Neo, Hi	Not associated with *E. globulus*
Laccaria bicolor (DQ367906)	98	759	Ep, Q<br>S, H	KY655010	Nea, Neo, Pal, Hi, Aus	Not associated with *E. globulus*
Laccaria lateritia (JX270751) [6]	98	423	Ep, E	KY655011	Pal, Neo, Hi, Aus	Co-introduced

Lactarius camphoratus (DQ422009)	98	747	Qf, E	KY659396	Pal, Nea, Neo, Hi	Novel[7]
Lactarius hepaticus (AY254881)	99	616	Pp, P	KY659395	Pal, Nea, Aus	Not associated with *E. globulus*
Lactarius luculentus (FJ845419)	98	822	Pp, P	KY659407	Nea	Not associated with *E. globulus*
Lactarius obscuratus (HQ714739)	98	791	Pp, P	KY659397	Pal, Nea, Neo, Hi	Not associated with *E. globulus*
Lactarius quietus (KR025623)	100	809	Qf, Q<br>Ep, Q	KY659408	Pal, Nea, Neo, Hi, Aus	Not associated with *E. globulus*
Lactarius subdulcis *(AJ889963)*	98	817	Qf, Q<br>Qf, E<br>Pp, P<br>S, H	KY681468	Pal, Nea, Neo, Hi, Aus	Putative novel [7]
Lactarius tabidus (KP783447)	98	592	Qf, E	KY681466	Pal, Nea	Novel[7]
Paxillus involutus (FR750011)	99	873	Qf, Q	KY681467	Pal, Nea, Neo, Hi, Aus	Not associated with *E. globulus*
Pseudotomentella sp. (GQ267479)	99	738	Ep, P<br>Pp, P<br>Pp, E	KY681803	Pal, Nea, Neo, Afr, Hi Aus*	Novel[8]
Pseudotomentella tristis (AJ889979)	99	724	Ep, P<br>Pp, P	KY681465	Nea, Pal, Hi	Not associated with *E. globulus*
Russula sp. (JN129409)	97	690	Qf, Q<br>Qf, E<br>Pp, P<br>Ep, Q<br>Ep, P	KY694395	Pal, Nea, Neo, Afr, Hi Aus	Undetermined[3]
Russula amoenolens (KF245510)	100	763	Qf, E	KY681463	Nea, Neo, Pal, Aus*	Novel[2]
Russula bicolor (HQ604845)	98	875	Pp, P	KY681462	Nea, Hi	Not associated with *E. globulus*
Russula drimeia (AY061672)	99	856	Pp, P	KY681464	Nea, Pal, Aus	Not associated with *E. globulus*
Russula fragilis (KM576547)	98	620	Ep, Q	KY681458	Nea, Neo, Pal, Hi, Aus	Not associated with *E. globulus*
Russula sp.<br>*Russula emetica* (AY228360)	96	756	Pp, P	KY693662	Pal, Nea, Neo, Afr, Hi Aus	Not associated with *E. globulus*
Russula laccata (HQ604844)	98	857	Pp, P	KY681459	Nea, Pal, Aus	Not associated with *E. globulus*
Russula nigricans (KC5881314)	99	779	Qf, Q	KY681455	Pal, Nea, Neo, Hi, Aus	Not associated with *E. globulus*
Russula ochroleuca (AY254880)	99	807	Ep, Q<br>Qf, E	KY681456	Nea, Pal, Hi	Novel
Russula parazurea (DQ422007)	98	691	Pp, P	KY681457	Nea, Neo, Pal	Not associated with *E. globulus*
Russula puellula (JF908706)	99	798	Qf, Q	KY693660	Pal	Not associated with *E. globulus*
Russula sardonia (JQ888201)	99	844	Pp, P	KY693646	Nea, Neo, Pal, Afr	Not associated with *E. globulus*

Russula violeipes (GU371290)	98	575	Pp, P	KY693648	Nea, Pal, Hi	Not associated with *E. globulus*
Scleroderma citrinum (HM189957)	99	783	Ep, Q<br>Ep, P<br>Ep, H<br>S, H<br>Pp, P	KY694393	Pal, Nea, Neo, Hi Aus	Not associated with *E. globulus*
Scleroderma polyrhizum (EU718123)	98	700	Qf, E<br>Ep, H	KY693661	Nea, Neo, Hi, Pal, Aus*	Novel[9]
Sebacina sp. (AB831807)	99	584	Pp, P	KY694396	Pal, Nea, Neo, Hi, Aus	Not associated with *E. globulus*
Suillus bovinus (AY898623)	99	778	Ep, P<br>Pp, P	KY693685	Pal, Nea, Neo, Hi, Aus	Not associated with *E. globulus*
Thelephora terrestris (JQ711981)	99	748	Ep, Q<br>Ep, H<br>Pp, P<br>S, H	KY693686	Pal, Nea, Hi, Aus, Afr	Not associated with *E. globulus*
Tylospora sp. (KF007260)	90	490	Qf, E<br>Qf, Q	KY711370	Pal, Nea	Novel[10]
Tomentella sp. (JQ888215)	90	797	Qf, E<br>Qf, Q	KY694394	Pal, Nea, Neo, Hi, Afr, Aus	Undetermined[3]
Tomentella badia (JQ711987)	98	516	Ep, Q<br>Qf, Q<br>S, H	KY693714	Nea, Neo, Pal, Hi	Not associated with *E. globulus*
Tomentella stuposa (AY635168) [12]	99	806	Qf, E<br>Qf, Q<br>Ep, Q	KY693712	Pal, Nea, Neo, Hi, Afr, Aus	Putative novel [11]
Tomentella sublilacina (KP783476)	98	748	Qf, E<br>Qf, Q<br>Ep, Q	KY693713	Pal, Nea, Neo, Afr, Aus	Putative novel [11]
Tomentellopsis submollis (AY641459)	98	856	Ep, H<br>S, H<br>Pp, P<br>Ep, P	KY693726	Pal, Nea	Not associated with *E. globulus*
Xerocomus badius (HQ207696)	99	679	Qf, E<br>Pp, P<br>Ep, P	KY693727	Pal, Nea, Neo, Hi	Novel
Xerocomus chrysenteron (HQ207694)	98	836	Qf, Q	KY693968	Nea, Neo, Pal, Hi, Aus	Not associated with *E. globulus*
Xerocomus rubellus (JQ685725)	98	924	Qf, Q	KY693966	Nea, Pal, Aus	Not associated with *E. globulus*
Xerocomus subtomentosus (DQ066365)	100	677	Qf, Q	KY693967	Nea, Neo, Pal, Hi, Aus	Not associated with *E. globulus*

[1]Sequence similarity to *Amanita gemmata* was high (94%) but did not reach the 98% threshold for species identification.
[2] *Amanita gemmata* has been recorded in the Australasian region, but only in New Zealand, where *E. globulus* is an exotic species (Whiters, 2001). For this reason, its association with *E. globulus* was classified as novel.
[3]This genus has numerous observations in both Europe and Australia (see at www.gbif.org), making it difficult to determine their native region and thus the type of interaction it establishes with *E. globulus*.
[4]*Cenococcum geophilum* is a cosmopolitan ECM fungus in the Holarctic realm (see at www.gbif.org) with a high adaptability to different habitats and host plants (Lo Buglio et al. 1996). Trappe (1962) showed the presence of *C. graniforme*, synonym of *C. geophilum*, mainly in the Northern Hemisphere. He also mentions the first discovery of the species in the Southern Hemisphere, in South Africa, in Stellenbosch. In regard to Australia, however, Trappe (1962) explicitly states that the species had not been encountered in Australia or New Zealand by researchers working with mycorrhizae. This leads us to conclude that recent citations of the species in Australia (Tedersoo et al. 2008, see also http://www.ala.org.au/blogs-news/fungimap-putting-australian-fungi-on-the-map/) probably correspond to new introductions there. For this reason, the association between this species and *E. globulus* was classified as putative novel.
[5] *Cortinarius anomalus* has one record in Australia, but in the western region, where *E. globulus* is not native. For this reason, its association with *E. globulus* was classified as novel.
[6]*Laccaria lateritia* is sometimes synonymized with *L. fraterna*, see at www.mycobank.org.
[7]Despite the genus *Lactarius* was generally considered not compatible with eucalypt roots in pure culture synthesis (Malajczuk et al. 1982) it was found under *Eucalyptus* spp. in the field in Tasmania (Tedersoo et al. 2008; Gates et al. 2011a; Gates et al. 2011b; Horton et al. 2013). In the present study we detected three species never described before in association with *E. globulus*.
[8]There are very few and recent observations of *Pseudotomentella* sp. in Australia, but not in the native area of *E. globulus*.
[9]*Scleroderma polyrhizum* has been considered native to the Northern Hemisphere by Vellinga et al. (2009), despite it has been frequently recorded in NE Australia, outside the native range of *E. globulus*. For this reason, its association with *E. globulus* was classified as novel.
[10]*Tylospora* sp. has no records in Australia, for this reason it was classified as novel.
[11]The *Tomentella* genus is a common member of the fungal community, although little information exists on its host specificity. This genus is present in Australia with *T. brunneorufa* Larsen (Kirk et al. 2010; Agerer & Bougher, 2001), and in Tasmania with several species (Tedersoo et al. 2008), in association with *Eucalyptus*.
[12] *Tomentella stuposa* was detected in Tasmania by Tedersoo et al. (2008) in a forest dominated by *E. regnans*. This is the first time on our knowledge that this species is reported in association with *E. globulus*.

References

Agerer R. & Bougher N.L. *Tomentella brunneorufa* M. J. Larsen + *Eucalyptus* spec. *Descr. Ectomyc*. 5: 205-212 (2001).

Bergero, R., Perotto, S., Girlanda, M., Vidano, G. & Luppi, A. M. Ericoid mycorrhizal fungi are common root associates of a Mediterranean ectomycorrhizal plant (*Quercus ilex*). *Mol. Ecol.* 9: 1639–1649 (2000).

Dickie, I. A. *et al.* The emerging science of linked plant-fungal invasions. *New Phytol* 215:1314-1332 (2017).

Gates, G.M., Mohammed, C., Ratkowsky, D., Wardlaw, T. & Davidson N.J. Diversity and ecology of epigeous ectomycorrhizal macrofungal assemblages in a native wet eucalypt forest in Tasmania, Australia. *Fungal Ecol.* 4: 290-298. (2001a)

Gates, G.M., Mohammed, C., Wardlaw, T, Davidson N.J. & Ratkowsky, D.A. Diversity and phenology of the macrofungal assemblages supported by litter in a tall, wet *Eucalyptus obliqua* forest in southern Tasmania, Australia. *Fungal Ecol*. 4: 68-75 (2011b).

Horton, B.M., Glen, M., Davidson, N.J., Ratkowsky, D., Close D.C., Wardlaw, T.J., & Mohammed, C. Temperate eucalypt forest decline is linked to altered ectomycorrhizal communities mediated by soil chemistry. *Forest Ecol. Manag.* 302: 329-337 (2013).

Kirk P.M., Cannon, P. F., Minter, D. W. & Stalpers, J. A. 2010. Dictionary of the Fungi (10th ed.). Wallingford, UK: CAB International. p. 201

Lo Buglio, K.F., Berbee, M.L., Taylor, J. W. Phylogenetic origins of the asexual mycorrhizal symbiont *Cenococcum geophilum* Fr. and other mycorrhizal fungi among the Ascomycetes. *Molecular Phylogenetics and Evolution* 6: 287-294 (1996).

Malajczuk, N., Molina, R. & Trappe, J.M. Ectomycorrhiza formation in *Eucalyptus*. I. Pure culture synthesis, host specificity and mycorrhizal compatibility with *Pinus radiata*. *New Phytol*. 91:67-482 (1982).

Tedersoo L., et al. Ascomycetes associated with ectomycorrhizas: molecular diversity and ecology with particular reference to the Helotiales. *Environ. Microbiol.* 11: 3166-3178 (2009).

Tedersoo, L., Jairus, T., Horton, B.M., Abarenkov, K., Suvi, T., Saar, I., Koljalg, U. Strong host preference of ectomycorrhizal fungi in a Tasmanian wet sclerophyll forest as revealed by DNA barcoding and taxon-specific primers. *New Phytol.* 180, 479-490 (2008).

Trappe, J. M. ‘‘*Cenococcum graniforme*—Its Distribution, Ecology, Mycorrhiza and Inherent Variation,’’ Ph.D. Dissertation, University of Washington, Seattle, WA. (1962).

Vellinga, E. C., Wolfe, B. E. & Pringle, A. Global patterns of ectomycorrhizal introductions. New Phytol. 181: 960-973 (2009).

Withers, T. M. Colonization of eucalypts in New Zealand by Australian insects. Austral Ecol. 26: 467-476 (2001).

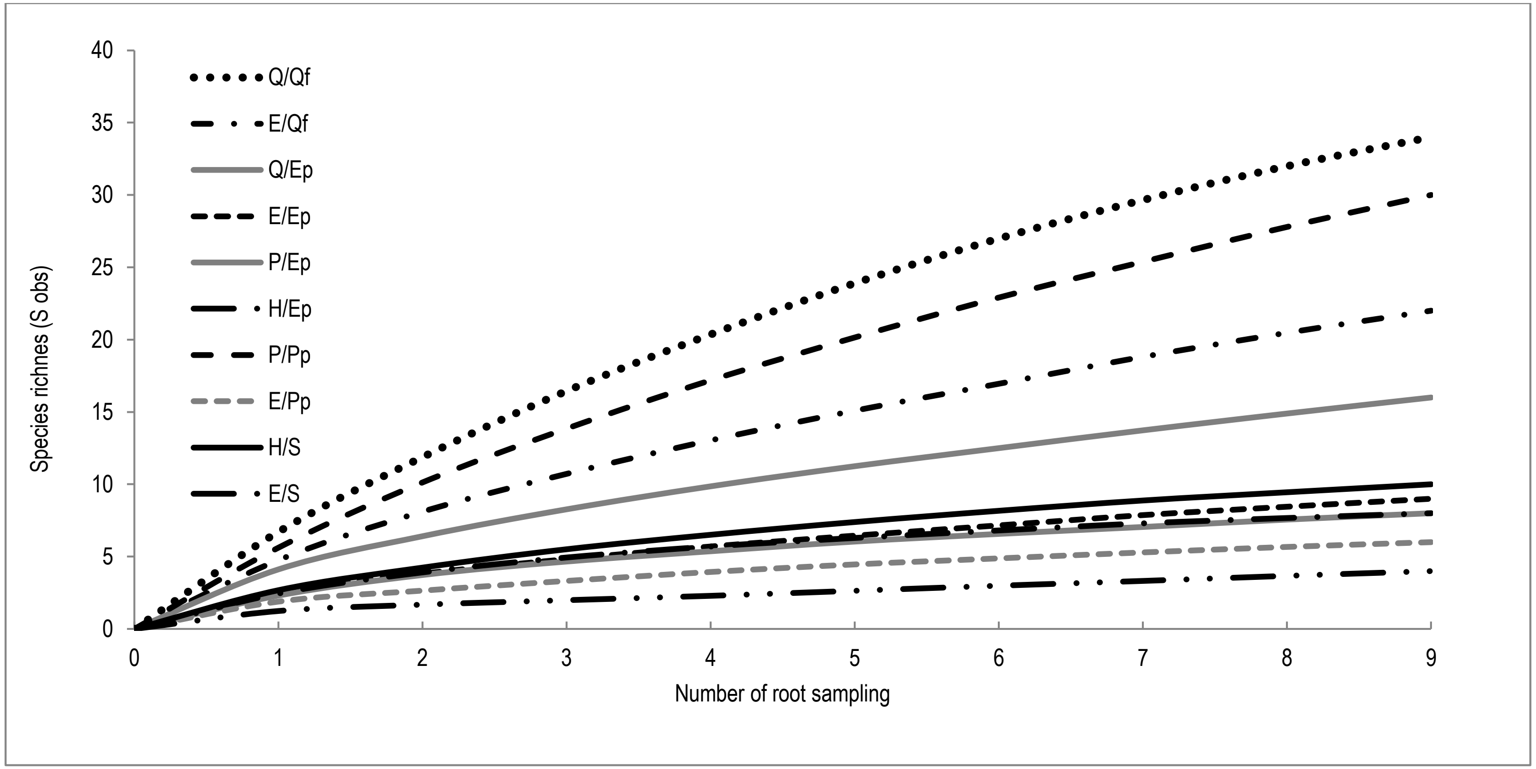

Supplementary Figure S1. Rarefied species accumulation curves of four plant species recorded in four types of habitat (beta diversity). Each curve represents the mean of 500 randomizations of sampling order. *Eucalyptus globulus* (E), *Pinus pinaster* (P), *Quercus robur* (Q) and *Halimium lasianthum* (H) in four habitats (*E. globulus* plantation = Ep; *P. pinaster* plantation = Pp; *Q. robur* forest = Qf; and shrubland = S).

Supplementary Table S3. Observed (S_{obs}) and estimated (S_{est}, with the Chao2 method) species richness and the ratio between them. Abbreviations as per figure S1.

	Q/Qf	P/Pp	E/Qf	Q/Ep	H/S	E/Ep	H/Ep	P/Ep	E/Pp	E/S
S_{obs}	34	30	22	16	10	9	8	8	6	4
S_{est}	46	59.63	43.78	38.22	10	14.56	10	15.11	10	6.67
S_{est}/S_{obs}	0.739	0.503	0.503	0.419	1.000	0.618	0.800	0.529	0.600	0.600

Supplementary Table S4. Results of the PERMANOVA analysis to test for differences in ECM fungal taxa composition between host plant species within habitats (all the studied combinations of plant species in each habitat).

**Source**	**df**	**SS**	**MS**	**Pseudo-F**	**P (perm)**
Site	2	5363.9	2681.9	1.0951	0.3282
Plant species/Habitat	9	1.3349E+05	14833	5.509	0.0001
Site x Plant species/Habitat	18	48464	2692.4	1.0994	0.1652
Res	60	1.4694E+05	2448.9		60
Total	89	3.3426E+05		Total	89

Supplementary Table S5. Pairwise comparisons between host plant species within habitats in ECM fungal taxa composition. Average percentage similarities and significance of the difference between cases are shown in each cell, with P values symbolized as * for P≤.05, ** for P≤.01, *** for P≤.001 and ns for non-significant differences. *Eucalyptus globulus* (E), *Pinus pinaster* (P), *Quercus robur* (Q) and *Halimium lasianthum* (H) in four habitats (*E. globulus* plantation = Ep; *P. pinaster* plantation = Pp; *Q. robur* forest = Qf; and shrubland = S).

Host plant/Habitat	Q/Qf	E/Qf	Q/Ep	E/Ep	H/Ep	P/Ep	E/S	H/S	E/Pp	P/Pp
Q/Qf	35,152									
E/Qf	23,984*	24,437								
Q/Ep	32,755 ns	25,896 ns	34,797							
E/Ep	10,018**	23,325*	16,482**	33,141						
H/Ep	0,26101**	14,072**	6,2377**	31,862 ns	33,002					
P/Ep	13,558**	22,746 ns	20,231**	24,946 ns	18,598**	26,382				
E/S	7,6541E-2**	20,389**	7,7648**	40,159 ns	42,084 ns	23,491**	52,813			
H/S	1,1576**	1,7586**	5,3216**	2,8472**	6,4173**	1,5915**	3,193**	27,006		
E/Pp	4,0292 **	28,804**	13,673**	42,98 ns	37,113**	26,905**	54,57 ns	1,6939**	61,861	
P/Pp	4,5714**	3,9826**	1,7002**	0,70488**	0,21108**	5,1649**	1,57E-2**	4,0085**	0,44117**	14,028
